# Parental obesity and risk factors for cardiovascular disease among their offspring in mid-life: findings from the 1958 British Birth Cohort Study

**DOI:** 10.1038/ijo.2013.40

**Published:** 2013-04-09

**Authors:** R Cooper, S M Pinto Pereira, C Power, E Hyppönen

**Affiliations:** 1 MRC Unit for Lifelong Health and Ageing, University College London, London, UK; 2 MRC Centre of Epidemiology for Child Health, Centre for Paediatric Epidemiology and Biostatistics, UCL Institute of Child Health, University College London, London, UK

**Keywords:** intergenerational associations, cardiovascular risk factors, BMI, cohort study, life course

## Abstract

**Background:**

Few studies have investigated whether parental adiposity is associated with offspring cardiovascular health or the underlying pathways. Studying these associations may help to illuminate the paradox of increasing prevalence of obesity and declining trends in cardiovascular disease (CVD) mortality, which may be partially explained by beneficial adaptations to an obesogenic environment among people exposed to such environments from younger ages.

**Objective:**

To investigate associations between parental body mass index (BMI) and risk factors for CVD among their offspring in mid-life and to test whether associations of offspring BMI with CVD risk factors were modified by parental BMI.

**Methods:**

Data from parents and offspring in the 1958 British birth cohort were used (*N*=9328). Parental BMI was assessed when offspring were aged 11 years; offspring BMI, waist circumference and CVD risk factors (lipid levels, blood pressure, glycosylated haemoglobin (HbA1c) and inflammatory and haemostatic markers) were measured at 44–45 years.

**Results:**

Higher parental BMI was associated with less favourable levels of offspring risk factors for CVD. Most associations were maintained after adjustment for offspring lifestyle and socioeconomic factors but were largely abolished or reversed after adjustment for offspring adiposity. For some CVD risk factors, there was evidence of effect modification; the association between higher BMI and an adverse lipid profile among offspring was weaker if maternal BMI had been higher. Conversely, offspring BMI was more strongly associated with HbA1c if parental BMI had been higher.

**Conclusions:**

Intergenerational influences may be important in conferring the effect of high BMI on CVD risk among offspring.

## Introduction

Associations between parental and offspring adiposity have consistently been shown with recent evidence demonstrating that these associations persist into mid-adulthood,^1, 2^ when risk factors for cardiovascular disease (CVD) are reaching clinically significant levels and symptoms of CVD are beginning to manifest in an increasing proportion of the population. Given the parental–offspring adiposity associations that persist across life,^1, 2^ the strong link between obesity and CVD risk^3, 4^ and the familial aggregation of genetic and environmental risk factors for obesity and CVD,^5, 6, 7, 8, 9^ parental adiposity may also influence offspring CVD risk. However, few studies have investigated whether parental adiposity is associated with offspring cardiovascular health or the underlying pathways^10, 11, 12, 13^ and mostly these do not examine offspring beyond adolescence.^11, 12, 13^

Exploring the intergenerational associations between parental adiposity and offspring CVD risk may help to illuminate the paradox of increasing prevalence of obesity and declining trends in CVD mortality.^14^ Despite increases in average body mass index (BMI) and prevalence of obesity,^15^ CVD mortality rates and population levels of related risk factors such as blood pressure (BP) and cholesterol have declined over the same time period.^15, 16, 17, 18, 19, 20^ Some studies have shown that the strength of the associations between obesity and risk factors for CVD is weaker in birth cohorts born more recently than in cohorts born earlier in the twentieth century.^18, 19, 21^ Opposing trends for BMI and CVD risk, and the weakening of obesity–CVD associations may be largely explained by increases in medication use and favourable changes in lifestyles.^14, 18, 19, 20, 21^ Another possibility is that, because of exposure from earlier ages, fetal programming or other transgenerational effects, more recent generations could be better adapted to an obesogenic environment than older generations.^19^ Potentially, such adaptations might lead to lower risk of CVD in later life. It could also be expected that, if such adaptations are occurring, then among a particular generation the association between BMI and risk factors for CVD in adulthood would be weaker among those whose parents were obese during their childhood than those whose parents were not obese.

Using intergenerational data from the 1958 British Birth Cohort Study, we aimed to establish: (1) associations between parental BMI and a comprehensive range of risk factors for CVD among their offspring in mid-life; (2) whether associations were explained (that is, mediated or confounded) by lifestyle and socioeconomic factors or by the parental–offspring adiposity association; (3) whether parental BMI modifies the associations between offspring BMI and risk factors for CVD, specifically whether associations are weaker among individuals whose parents had a higher BMI than among those whose parents had a lower BMI.

## Materials and methods

The 1958 British birth cohort, who constitute the offspring in our study, originally included all those born during 1 week in March 1958 across England, Scotland and Wales (*n*=17 638).^22^ Surviving cohort members were followed up into adulthood, with additional recruitment of 920 immigrants with the same birth dates into the study up to age 16 years. A target sample of 11 971 cohort members was invited to participate in a biomedical survey at age 44–45 years, 9377 (78%) responded. Respondents were broadly representative of the total surviving cohort.^23^ Ethical approval has been obtained for this study and study participants have provided informed consent.

### Offspring CVD risk factors

All risk factors for CVD among the offspring were measured at age 44–45 years. BP was measured three times after the participant had been seated for 5 min using an Omron 705CP automated digital oscillometric sphygmomanometer (Omron, Tokyo, Japan); a large cuff was used if the mid-upper arm circumference was >32 cm. Mean diastolic and systolic BP values were used.

Non-fasting venous blood samples were obtained by nurses using standardized protocols during home visits and posted to central laboratories. Glycosylated haemoglobin (HbA1c) levels were measured using ion exchange high-performance liquid chromatography. Total and high-density lipoprotein (HDL)-cholesterol and triglyceride levels were measured by an autoanalyzer (Olympus AU640, Olympus, Tokyo, Japan) using enzymatic methods. Low-density lipoprotein (LDL)-cholesterol levels were calculated using the Friedewald formula^24^ except when triglyceride level >4.5 mmol l^–1^. Fibrinogen levels were measured using the Clauss method^25^ and C-reactive protein (CRP) assayed by nephelometry (Dade Behring) on citrated plasma samples after one thaw cycle. von Willebrand factor (vWF) antigen was measured by Decollates enzyme-linked immunosorbent assay (ELISA) and tissue plasminogen activator (t-PA) antigen by Biopool ELISA. Fibrin D-dimer was measured on stored samples at the end of the data collection period by ELISA assay (Hyphen, Paris, France) and standardized for inter-batch variation.

As ignoring or excluding participants using medication can bias estimates of association,^26^ correction was made for medication use. Nurses obtained information on currently prescribed medication through direct observation of packaging from which lipid-lowering drugs (for example, statins) and medications for hypertension and diabetes were identified. A commonly used constant of 10 mm Hg, which has been suggested on the basis of evidence from clinical trials, was added to the measures of diastolic and systolic BP for those on treatment for hypertension (*n*=424).^26^ For those taking oral antidiabetic medication for type 2 diabetes (*n*=107), a correction was made to HbA1c levels on the assumption that their medication reduced HbA1c levels by 1% in absolute terms.^27^ Lipid levels of those on lipid-lowering drugs (*n*=162) were corrected to allow for effects of treatment assuming that lipid-lowering drugs reduce total cholesterol by 20%, LDL-cholesterol by 35%, triglycerides by 15% and increase HDL-cholesterol by 5%, based on average efficacy of a statin, the most frequently prescribed lipid-lowering drug in this study.^28^

### Offspring BMI and waist circumference

BMI (kg m^–2^) was calculated using height and weight measurements, taken using Leicester portable stadiometers and Tanita solar scales, respectively, by nurses using standardized protocols while participants were lightly clothed and unshod. Self-reported weights (*n*=80) and heights (*n*=68) were used if measurements were inaccurate or consent for measurement was not provided. Waist circumference (cm) was measured midway between the lower ribs and iliac crest.

### Parental BMI

Parents' heights and weights were reported in 1969 when their offspring were aged 11 years. Heights were reported in feet and inches to the nearest inch. Weights were reported in pre-classified groups ranging from 6 stone 4 pounds (39.9 kg) to 19 stone 10 pounds (125.2 kg) in increments of 6 pounds (2.7 kg). To calculate BMI, heights were converted into metres and a weight in kg was assigned, which was equivalent to the midpoint of the recorded weight category. For some analyses, maternal and paternal BMI were categorized into four standard groups: <20 (underweight); 20–25 (normal); 25–30 (overweight); and >30 kg m^–2^ (obese).

### Covariates

Key variables that could mediate or confound the main associations of interest were selected *a priori*. Some covariates, notably lifestyle factors, had been assessed at different adult ages and so, to take into account the potential cumulative effects or changes in these factors across adulthood, we included information from two ages (one in earlier and one later in adulthood) in analyses. A detailed description of covariates is reported elsewhere^1^ and so a brief description follows. Parental ages were recorded at the time of their offspring's birth. At ages 33 and 42 years, offspring reported their frequency of consumption of different food groups. Using this information, variables were created to identify the amount of fried food and fruit consumed as indicators of overall diet. Physical activity was ascertained by self-report of frequency of participation in sports or other regular physical activity at ages 23 and 42 years and sedentary behaviour was based on self-reports of hours per day spent watching television at ages 23 and 44–45 years. Smoking status was self-reported at ages 23 and 42 years (or at 33 years if missing). Alcohol consumption at age 23 years was assessed from self-reports of alcoholic beverages consumed within the previous week and a quantity-frequency index of alcohol use at age 44–45 years was derived from the Alcohol Use Disorders Identification Test questionnaire.^29^

Lifetime socioeconomic position (SEP) was indicated by father's occupational class at birth (or at 7 years if missing) and offspring's educational level and own occupational class ascertained at age 42 years (or, for occupational class, at 33 years if missing). Both measures of occupational class were categorized into four groups using a standard method of categorizing occupations in the United Kingdom, the Registrar General's Social Classification: 1 I (professional) or II; 2 III non-manual; 3 III manual; and 4 IV, V (unskilled), or single mother. Educational level was categorized into five groups from no qualifications to degree or higher.

### Statistical analyses

The normality of the CVD risk factors was assessed and in cases where they were skewed (HbA1c, triglycerides, CRP and D-dimer) geometric mean values are presented in the descriptive table. For ease of interpretation and to maintain consistency across outcomes, all outcome variables were log-transformed and multiplied by 100, whereby the regression coefficients can be interpreted as the symmetric percentage difference in means.^30^

We assessed the associations of parental BMI with offspring risk factors for CVD using linear regressions with three levels of adjustment. First, we tested the direct associations between parental BMI and each offspring CVD risk factor adjusting only for parental age and offspring's gender (model 1). Second, we examined whether the associations were explained (mediated or confounded) by lifestyle or socioeconomic factors. This was done by adjusting for the offspring's lifestyle factors in adulthood (that is, fried food and fruit consumption at 33 and 42 years, television viewing at 23 and 44–45 years, physical activity at 23 and 42 years, smoking status at 23 and 42 years and alcohol consumption at 23 and 44–45 years) and lifetime SEP (model 2). Third, we examined the extent to which tracking of BMI across generations explained parental influences on offspring CVD risk by adjusting for offspring's own BMI and waist circumference (model 3). To evaluate whether there were gender specific effects, we carried out formal tests of gender interaction (adjustments as in model 1). If evidence for interaction was found all subsequent analyses were stratified by gender. In all models, parental BMI was initially included as a categorical variable. Tests of deviation from linearity were performed and where there was no evidence of this, parental BMI was included in a second set of models as a continuous term.

To establish whether parental BMI modified the association between offspring BMI and CVD risk, in further analyses we tested the interactions between offspring and parental BMI. In these analyses, both parental and offspring BMI were modelled as continuous variables, in association with the CVD risk factors of the offspring with adjustments for parental age and gender and subsequently also for offspring lifestyle factors and lifetime SEP. Where evidence of interaction was found we: (i) undertook stratified analyses (by tertiles of parental BMI) to characterize the underlying effect modification, and (ii) evaluated the possibility that offspring weight change between childhood and adulthood accounted for the interaction between parental and offspring BMI, by adjusting for offspring BMI at age 11 years.

All models described above were performed for maternal BMI and paternal BMI separately; that is, none of the models included both maternal and paternal BMI together. Analyses were run on a sample of up to 9328 offspring who had participated in the biomedical survey, were not pregnant (exclusions because of pregnancy *n*=2) and had valid data on at least one risk factor for CVD. Offspring with type 1 diabetes (*n*=55) were excluded from analyses of HbA1c. The number included in any one set of analyses varied between 7747 (for BP) and 6169 (for LDL-cholesterol) because of variation in missing outcome, offspring BMI and parental BMI data. To minimize the loss in numbers and potential bias introduced because of missing information, missing values for parental BMI and covariates were imputed using multiple imputation chained equations implemented in Stata version 11.^31^ The multiple linear regression analyses described above were run across 10 imputed data sets and results from these models are presented. The main models were re-run on the maximum available samples, leading to identical interpretation. Further sensitivity analyses were performed to examine the potential impact of postal delay of blood sample, month of examination, time of day, recent food consumption, air temperature and assay batch on the main findings, but as these factors had a negligible influence; related data are not shown.

## Results

At age 44–45 years, 25.4% of male and 23.8% of female offspring were classified as obese (that is, BMI >30 kg m^–2^) whereas when their offspring were aged 11 years, only 5.4% of fathers and 7.8% of mothers were obese. The correlation between maternal and paternal BMI was low (*r*=0.11). Obese offspring were more likely to have had an overweight or obese mother or father during their childhood and to have a less favourable CVD risk factor profile at age 44–45 years than offspring who were not obese (Table 1).

**Figure 1:**
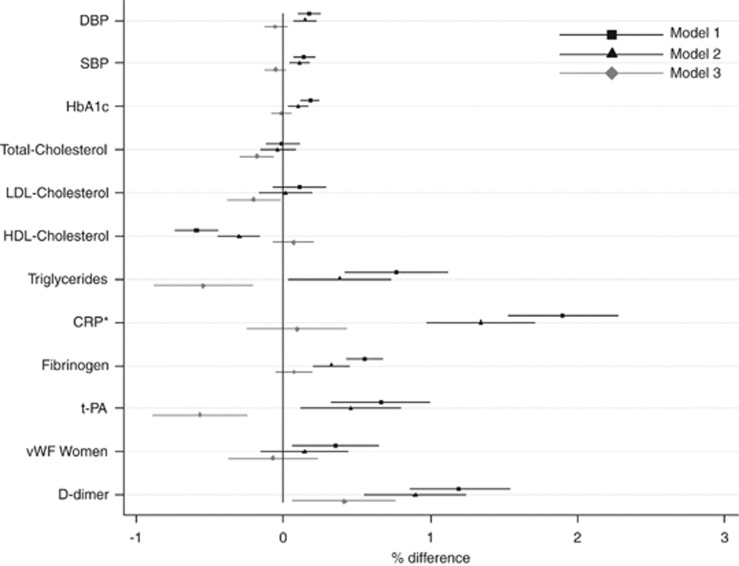
Percent differences in mean levels of offspring risk factors for CVD at age 44–45 years per 1 kg m^–2^ increase in maternal BMI (*N*=7747 (for BP) to 6169 (for LDL-cholesterol)). Model 1: adjusted for maternal age and gender (unless models are gender-stratified). Model 2: model 1 plus offspring lifestyle factors (fried food and fruit consumption at 33 and 42 years, television viewing at 23 and 44–45 years, physical activity at 23 and 42 years, smoking status at 23 and 42 years and alcohol consumption at 23 and 44–45 years) and lifetime SEP (father's and own occupational class and education level). Model 3: model 2 plus offspring BMI and waist circumference. The association between maternal BMI and vWF in men is not presented because of evidence of non-linearity (*P*-value for quadratic term in model 1=0.03, overall test of association *P*=0.11). *Percent increase in mean CRP levels of offspring at age 44–45 years per 0.5 kg m^–2^ increase in maternal BMI. Corresponding values per 1 kg m^–2^ increase in maternal BMI are: 3.79% (3.05%, 4.54%), 2.69% (1.95%, 3.42%) and 0.19% (−0.49%, 0.86%) for models 1, 2 and 3, respectively. DBP, diastolic BP; SBP, systolic BP.

In models adjusted for maternal age and offspring gender, higher maternal BMI was associated with lower levels of HDL-cholesterol and higher levels of all other outcome measures among offspring at age 44–45 years with the exception of total and LDL-cholesterol where no association was seen (Figure 1). The strongest association was between maternal BMI and CRP; per 1 kg m^–2^ increase in maternal BMI, CRP was higher by 3.8% (95% confidence interval) 3.1–4.5) after adjustment for gender and maternal age. There were no gender interactions for most outcomes, however, higher maternal BMI was associated linearly with higher vWF levels in female but not male offspring (*P*-interaction<0.01). In men, the association between maternal BMI and vWF levels was non-linear, with higher mean levels of vWF among male offspring of underweight compared with normal weight mothers (*P*=0.03 for quadratic term), hence data in relation to vWF for men are not presented in Figure 1. Most associations were attenuated slightly after adjustment for offspring lifestyle factors and indicators of lifetime SEP. After further adjustment for offspring adiposity, most associations were no longer found. However, for total and LDL-cholesterol, triglycerides and t-PA, associations were negative after adjustment for offspring adiposity.

**Figure 2:**
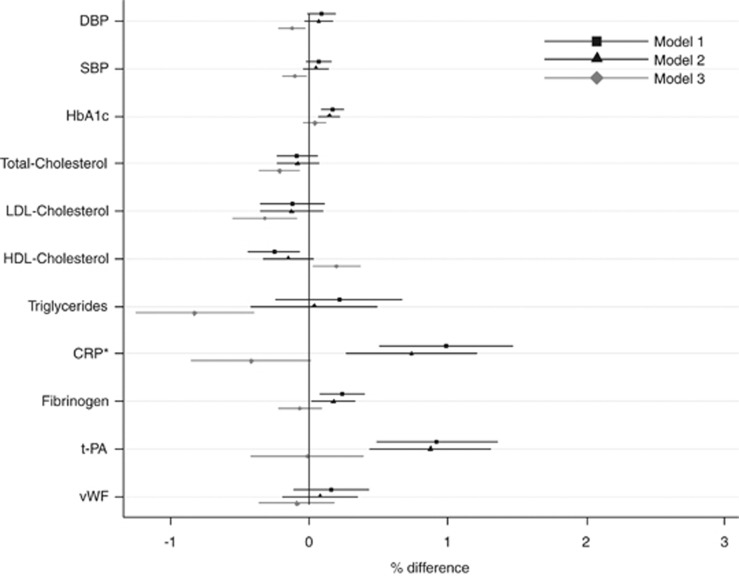
Percent differences in mean levels of offspring risk factors for CVD at age 44–45 years per 1 kg m^–2^ increase in paternal BMI (*N*=7533 (for BP) to 5979 (for LDL-cholesterol)). Model 1: adjusted for paternal age and gender. Model 2: model 1 plus offspring lifestyle factors (fried food and fruit consumption at 33 and 42 years, television viewing at 23 and 44–45 years, physical activity at 23 and 42 years, smoking status at 23 and 42 years and alcohol consumption at 23 and 44–45 years) and lifetime SEP (father's and own occupational class and education level). Model 3: model 2 plus offspring BMI and waist circumference. *Percent increase in mean CRP levels of offspring at age 44–45 years per 0.5 kg m^–2^ increase in paternal BMI. Corresponding values per 1 kg m^–2^ increase in paternal BMI are: 1.97% (1.01%, 2.94%), 1.46% (0.52%, 2.41%) and −0.84% (−1.70%, 0.02%) for models 1, 2 and 3, respectively. The association between paternal BMI and D-dimer is not presented because of evidence of non-linearity (*P*-value for quadratic term in model 1<0.01, overall test of association *P*<0.001). DBP, diastolic BP; SBP, systolic BP.

In models adjusted for paternal age and offspring gender, higher paternal BMI was associated with lower levels of HDL-cholesterol and higher HbA1c, CRP, fibrinogen and t-PA among offspring at age 44–45 years (Figure 2). As for maternal BMI, the strongest association observed was between paternal BMI and CRP; per 1 kg m^–2^ increase in paternal BMI, offspring CRP was higher by 2.0% (95% confidence interval 1.0–2.9, adjusted for gender and paternal age). Associations were attenuated after adjustment for offspring lifestyle factors and lifetime SEP, and after further adjustment for offspring adiposity the majority of associations were no longer found, although negative associations were seen with total and LDL-cholesterol, triglycerides and BP. There was no evidence of association between paternal BMI and vWF in any of the models. The association between paternal BMI and D-dimer levels was non-linear, with higher mean levels of D-dimer among offspring of underweight and overweight fathers compared with normal weight fathers (*P*<0.01 for quadratic term), hence this association is not presented in Figure 2.

**Figure 3:**
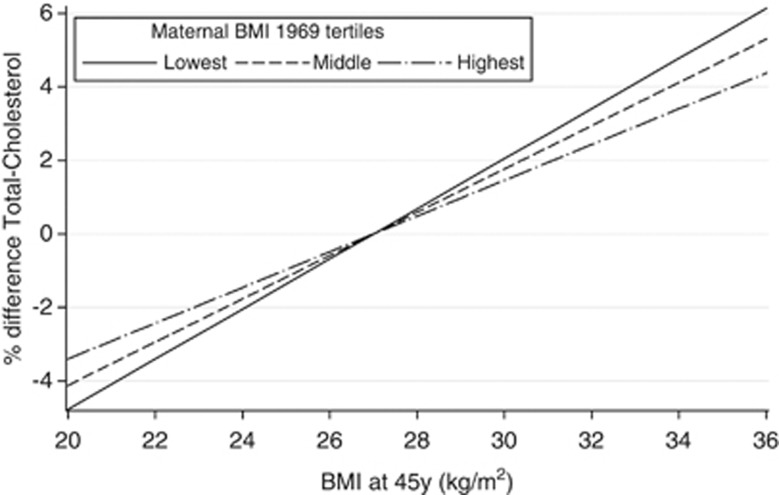
The association between offspring BMI (centred at 27 kg m^–2^) and percent difference in total cholesterol at age 44–45 years stratified by tertiles of maternal BMI. (*N*=2168, 2196 and 2175 for lowest, middle and highest stratum of maternal BMI, respectively).

**Figure 4:**
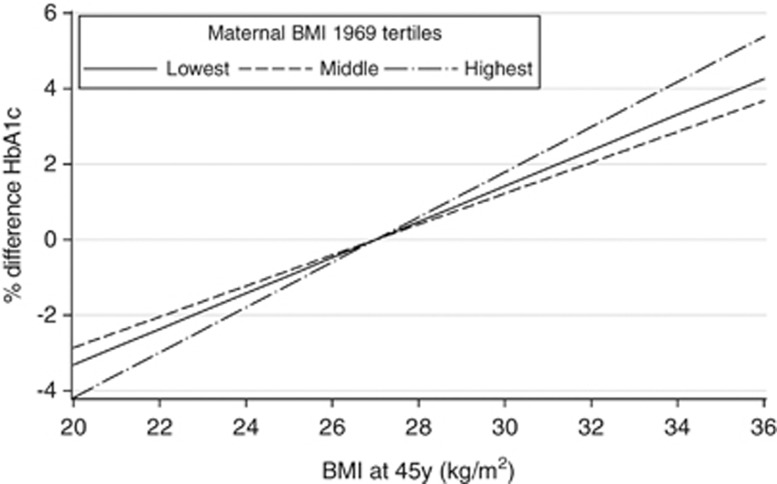
The association between offspring BMI (centred at 27 kg m^–2^) and percent difference in HbA1c at age 44–45 years stratified by tertiles of maternal BMI (*N*=2185, 2206 and 2188 for lowest, middle and highest stratum of maternal BMI, respectively).

In parental age and gender-adjusted models, maternal BMI was found to modify the association between offspring BMI and total cholesterol, LDL-cholesterol, triglycerides, HbA1c, systolic BP and CRP (*P*-interaction 0.003, 0.01, 0.002, 0.02, 0.02 and 0.04, respectively). In addition, there were interactions between paternal and offspring BMI for HbA1c and t-PA (*P*-interaction 0.01 and 0.04, respectively). All interactions remained after adjustment for lifestyle factors and lifetime SEP. When explored further, it was found that for all interactions except HbA1C, the positive associations of offspring BMI with the specified risk factors for CVD were weaker among those whose mothers had a higher BMI than among those whose mothers had a lower BMI. This is illustrated in Figure 3 for total cholesterol. For LDL-cholesterol and triglycerides, interactions appeared very similar to those for total cholesterol, whereas for systolic BP and CRP the patterns were less strong. In contrast, the positive association between BMI and HbA1c was stronger for offspring to mothers (or fathers) with higher compared with lower BMI (Figure 4). All interactions remained after adjustment for offspring BMI at age 11 years.

## Discussion

In a large nationally representative British birth cohort, participants whose parents had a higher BMI during their childhood generally had a more adverse CVD risk profile in mid-life than those whose parents had a lower BMI. Although the parental associations found were largely maintained after adjustment for offspring lifestyle and socioeconomic factors the majority were abolished or reversed after adjustment for offspring adiposity. However, for several of the risk factors for CVD, we found evidence for effect modification by intergenerational factors. Most notably the association between high BMI and adverse lipid profile, and to a lesser extent CRP and systolic BP, appeared to be weaker among offspring of mothers with higher BMI. In contrast, the association between BMI and HbA1c was stronger for offspring with a heavier parent.

### Comparison with other studies

Of the few studies to have investigated parental BMI and offspring levels of risk factors for CVD, all found some evidence of association.^10, 11, 12^ Furthermore, as in our study, associations with parental BMI were not fully consistent across all outcomes examined and, where tested, associations were attenuated after adjustment for offspring adiposity. In the only previous study to investigate parental obesity in relation to a range of outcomes associated with increased risk of CVD in offspring in mid-life, conducted in the USA, parental obesity was positively associated with CRP but not BP or diabetes.^10^ This study is consistent with our finding that the outcome most strongly associated with parental BMI before adjustment for offspring adiposity was CRP, suggesting an influence of obesity on low-grade inflammation. This American study^10^ also found that the offspring of two non-obese parents had higher total cholesterol levels than offspring with one or two obese parents, which is consistent with our finding of stronger associations between offspring BMI and lipid levels among those whose parents had lower BMI.

Our findings of a stronger association between offspring adiposity and HbA1c among those with heavier parents, is consistent with evidence that the strength of associations between obesity and diabetes risk have not reduced in younger compared with older generations in contrast to the associations between obesity and the majority of other CVD risk factors.^19^

### Explanation of findings

In mid-life, a more adverse CVD risk factor profile among offspring was found in association with greater adiposity of parents in basic models yet these associations attenuated after adjustment for offspring adiposity. This suggests that our findings are largely explained by the links between parental and offspring adiposity, which have been shown to be maintained into mid-life.^1, 2^ It is possible that some associations could be explained by greater weight gain among offspring of leaner parents. Further analyses conducted to explore this possibility (that is, adjustment for offspring BMI at 11 years) altered our findings very little, suggesting that maternal BMI—offspring CVD risk factor associations were not explained by offspring BMI gain. However, given previous observations in this population suggesting a role for offspring BMI gain on BP and HbA1c,^32, 33^ we cannot fully discount this possible explanation for observations reported.

It has also been proposed that maternal BMI could be associated with offspring CVD risk in later life as a result of programmed effects occurring in response to maternal nutrition *in utero*.^34^ If programming as a result of maternal under or overnutrition during pregnancy was a key explanation of our findings it would be expected that associations would be stronger for maternal adiposity assessed around the time of pregnancy. However, when analyses were rerun using mother's pre-pregnancy BMI findings generally resembled or were weaker than those presented (results not shown).

Our results showing weaker adult BMI and CVD risk factor associations for offspring of heavier mothers is consistent with beneficial adaptations occurring in response to exposure to an obesogenic environment. Such interactions were observed for several but not all CVD risk factors. Hence, the importance of adaptations that appear to be dampening the adverse effect of obesity on some pathways but not others and the mechanisms by which they could be occurring need to be investigated further, especially as such results could be due to chance. Interestingly, findings from tests of interaction between parental and offspring BMI suggest that the longer the exposure to an obesogenic environment the more detrimental the effects on glucose metabolism.

### Methodological considerations

There are a number of strengths to our study. These include the availability of a large sample, followed up to an age when CVD is beginning to manifest, that was selected to be nationally representative at birth and remains broadly so in mid-life.^23^ We were able to examine a wide range of outcomes all of which have been shown to be strongly associated with CVD risk,^35, 36, 37, 38, 39, 40, 41^ and adjust for several potential confounding and mediating factors across life, which were assessed prospectively. Further, in sensitivity analyses maternal smoking, which has been shown to be associated with offspring BMI and CVD risk,^42^ was also included and findings remained the same (results not shown). Including a wide range of covariates increased the number of participants with missing data on relevant measures but by running analyses on imputed data sets, bias that may have been introduced was minimized. Moreover, sensitivity analyses based on the maximum available samples showed similar findings.

Non-fasted blood samples were used to measure risk factors for CVD. Although total and HDL-cholesterol are not significantly affected by fasting status, triglyceride levels are lower after fasting^43^ and vary by duration of fasting and time of day.^44^ However, adjusting for time of blood collection and time since consuming food did not alter our findings. This along with other evidence including the positive correlation between fasting and non-fasting triglyceride levels^40, 45, 46^ suggests that use of non-fasting blood measures is likely to be acceptable for the purpose of our analyses. It has also been shown that there are important diurnal and seasonal variations in CRP, vWF, t-PA, fibrinogen and D-dimer levels.^47^ In sensitivity analyses, adjustments were made to allow for these variations and findings were unaltered.

Self-reported data on parental heights and weights may have resulted in some misclassification of parental BMI. Parents with higher weight might be expected to be more likely to under-report their weight, potentially leading to bias in our estimates towards the null. However, a key strength of our analyses was the prospective ascertainment of these data and the measurements for offspring. We have not compared findings for maternal and paternal BMI despite this being a potentially useful tool for discriminating between effects programmed *in utero* and other pathways. Similarly, we did not use a single exposure variable, which incorporates information on both parents' adiposity. There are a number of reasons for these decisions. First, paternal heights and weights were usually reported by mothers and so there may be more measurement error in father's anthropometric data than mother's. Second, we were unable to assess levels of non-paternity and third, combining information for both parents resulted in some very small categories (for example, both parents obese), which limited statistical power.

In our previous studies of intergenerational associations, we noted that parents of the 1958 cohort had lower rates of mortality than the general population.^1, 48^ However, as mortality differentials could be explained by lower mortality among parents than nulliparous people it is likely that parents of the 1958 cohort are representative of all parents in this generation. Our results do, however, need to be interpreted in context. Parents of the 1958 cohort had relatively low average BMI and only a small proportion were obese; with subsequent secular trends of increasing obesity, cohorts born more recently are more likely to have been exposed to obesogenic environments from younger ages. Thus, our findings may not be fully generalizable to younger cohorts. However, our finding that those who were obese but whose parents were not had, on average, the most adverse lipid profile is highly relevant and has important implications given that generations born more recently are more likely to have higher levels of adiposity than their parents.

### Implications

Our demonstration of associations between parental adiposity and offspring CVD risk adds to evidence suggesting that interventions to reduce population levels of obesity and CVD aimed at family units earlier in life are likely to be beneficial.^8, 9^ Associations appear to operate primarily through the tracking of obesity from one generation to the next suggesting that interventions should focus on weakening the link between parental and offspring adiposity. As the number of children born to parents with high BMI increases across the world it is of growing importance to elucidate the complex interplay between shared genetic and environmental factors, which underlie explanations of the associations between parental and offspring adiposity. Such knowledge could then be used to identify the most effective methods of intervention to weaken these links. Our study highlights the importance of breaking intergenerational links, not just to prevent the tracking of obesity from one generation to the next but also to reduce the impact on other offspring health outcomes including CVD. This is especially important for offspring glucose levels, which were higher if intergenerational tracking of obesity had occurred. There was, however, some suggestion that beneficial adaptations to an obesogenic environment had occurred in relation to lipid metabolism.

## Conclusions

Associations between parental BMI and offspring CVD risk factors in mid-life have been found and appear to be largely explained by the maintenance into adulthood of positive associations between parental and offspring adiposity. Some evidence of beneficial adaptations to an obesogenic environment are suggested by our findings and these appear to be specific to certain pathways. These novel findings require further investigation. Interventions to reduce population levels of overweight and obesity aimed at family units earlier in life are likely to be beneficial for many reasons including their impact on future CVD risk.

## Figures and Tables

**Table 1:** Characteristics of offspring at age 44–45 years stratified by their obesity status at this age (sample participating in the biomedical survey at age 44–45 years who had a measure of BMI at this age, *N*=4639 men and 4689 women)

	*Male offspring at 44–45 years*				*Female offspring at 44–45 years*	
	N^a^	*BMI⩽30 kg* *m*^–*2*^ *(Maximum* N*=3463) n (%) or mean (s.d.)*	*BMI>30 kg* *m*^–*2*^ *(Maximum* N*=1176) n (%) or mean (s.d.)*	N^a^	*BMI⩽30 kg* *m*^–*2*^ *(Maximum* N*=3575) n (%) or mean (s.d.)*	*BMI>30 kg* *m*^–*2*^ *(Maximum* N*=1114) n (%) or mean (s.d.)*
*Maternal BMI (kg* *m*^–*2*^*) in 1969*						
<20	463	396 (11.4)	67 (5.7)	488	412 (11.5)	76 (6.8)
20–25	2070	1618 (46.7)	452 (38.4)	2182	1758 (49.2)	424 (38.1)
25–30	1016	689 (19.9)	327 (27.8)	956	652 (18.2)	304 (27.3)
>30	309	183 (5.3)	126 (10.7)	301	169 (4.7)	132 (11.9)
Unknown	781	577 (16.7)	204 (17.4)	762	584 (16.3)	178 (16.0)
						
*Paternal BMI (kg* *m*^–*2*^*) in 1969*						
<20	139	113 (3.3)	26 (2.2)	151	118 (3.3)	33 (3.0)
20–25	2059	1634 (47.2)	425 (36.1)	2063	1639 (45.9)	424 (38.1)
25–30	1383	983 (28.4)	400 (34.0)	1368	1003 (28.1)	365 (32.8)
>30	201	103 (3.0)	98 (8.3)	205	136 (3.8)	69 (6.2)
Unknown	857	630 (18.2)	227 (19.3)	902	679 (19.0)	223 (20.0)
						
*Offspring adiposity and risk factors for cardiovascular disease at age 44–45 years*						
BMI (kg m^–2^)	4639	25.9 (2.5)	33.6 (3.6)	4689	24.5 (2.8)	35.1 (4.7)
Waist circumference (cm)	4621	94.1 (7.7)	111.4 (9.9)	4661	80.4 (8.3)	102.4 (10.7)
Diastolic blood pressure (mm Hg)	4627	80.9 (10.2)	85.6 (10.1)	4659	74.3 (10.0)	80.1 (9.8)
Systolic blood pressure (mm Hg)	4627	131.3 (14.6)	137.6 (15.1)	4659	118.5 (15.0)	126.3 (16.0)
HbA1c (%)^b^^c^	3935	5.2 (5.2, 5.2)	5.4 (5.4, 5.5)	3914	5.1 (5.1, 5.1)	5.4 (5.3, 5.4)
Total cholesterol (mmol l^–1^)	3916	6.0 (1.1)	6.2 (1.2)	3889	5.6 (1.0)	5.9 (1.1)
LDL-cholesterol (mmol l^–1^)	3555	3.5 (0.9)	3.6 (1.0)	3810	3.2 (0.9)	3.5 (0.9)
HDL-cholesterol (mmol l^–1^)	3903	1.5 (0.3)	1.3 (0.3)	3886	1.8 (0.4)	1.5 (0.3)
Triglycerides (mmol l^–1^)^b^	3901	1.9 (1.9, 2.0)	2.7 (2.6, 2.8)	3879	1.3 (1.2, 1.3)	1.8 (1.7, 1.9)
CRP (mg l^–1^)^b^	3845	0.8 (0.8, 0.9)	1.7 (1.6, 1.8)	3828	0.8 (0.7, 0.8)	2.9 (2.7, 3.1)
Fibrinogen (g l^–1^)	3834	2.8 (0.6)	3.0 (0.6)	3830	2.9 (0.6)	3.4 (0.7)
t-PA (ng ml^–1^)	3834	5.5 (2.8)	7.1 (2.7)	3815	4.0 (2.4)	6.0 (2.8)
vWF (IU dl^–1^)	3845	121.4 (40.3)	131.0 (41.8)	3829	118.0 (39.7)	131.6 (43.3)
D-dimer (ng ml^–1^)^b^	3824	133.8 (131.1, 136.6)	149.5 (144.6, 154.6)	3808	179.0 (175.5, 182.5)	231.9 (224.5, 239.5)

Abbreviations: BMI, body mass index; CI, confidence interval; CRP, C-reactive protein; HbA1c, glycosylated haemoglobin; HDL, high-density lipoprotein; LDL, low-density lipoprotein; t-PA, tissue plasminogen activator; vWF, von Willebrand factor.

a Total *N* varies because of variation in the amount of missing data.

b Geometric means and 95% CIs.

c In all, 55 type 1 diabetics excluded.
